# Prevalence of hepatitis B virus core antibodies among blood donors in Nigeria: Implications for blood safety

**DOI:** 10.4102/ajlm.v11i1.1434

**Published:** 2022-07-26

**Authors:** Foluke A. Fasola, Adeola A. Fowotade, Adedayo O. Faneye, Adeyeni Adeleke

**Affiliations:** 1 Department of Haematology, College of Medicine, University of Ibadan, Ibadan, Nigeria; 2 Department of Haematology, University College Hospital, Ibadan, Nigeria; 3 Department of Medical Microbiology & Parasitology, College of Medicine, University of Ibadan, Ibadan, Nigeria; 4 Department of Virology, College of Medicine, University of Ibadan, Ibadan, Nigeria

**Keywords:** anti-HBc antibodies, donors, blood safety, HBV DNA, occult HBV

## Abstract

**Background:**

Anti-hepatitis B core antibody (anti-HBc) testing improves transfusion safety by detecting past and current hepatitis B virus (HBV) infection while detecting hepatitis B surface antigen (HBsAg) in serology-negative HBV infection. However, occult HBV infection (OBI) (serum or liver HBV DNA-positive but HBsAg-negative) remains unaddressed among replacement blood donors – family members or friends who donate to replace blood transfused to a relative.

**Objective:**

This study assessed risk factors for a positive anti-HBc test among donors with OBI and determined the anti-HBc-positive status of replacement donors.

**Methods:**

The study was conducted at the University College Hospital Blood Bank, Ibadan, Nigeria, using blood samples collected from blood donors between April 2019 and May 2019. Donors were screened for HBsAg by rapid diagnostic test (RDT) and enzyme-linked immunosorbent assay (ELISA) and anti-HBc by ELISA, while HBV DNA was detected using a semi-nested polymerase chain reaction.

**Results:**

Of the 274 participants, 15 (5.5%) were HBsAg-positive by RDT and 36 (13.1%) by ELISA, while 133 (48.5%) were anti-HBc positive. Out of 232 HBsAg-negative donors, 107 (46.1%) were anti-HBc positive. Of the 107 HBsAg-negative but anti-HBc-positive samples, only one (0.93%) was HBV DNA-positive. The HBV DNA-positive donor was HBsAg-negative by both RDT and ELISA tests.

**Conclusion:**

This study establishes a potential risk for HBV transmission from isolated anti-HBc-positive donors to blood recipients. HBc immunoglobulin (antibody) M testing to identify blood units requiring further screening with polymerase chain reaction to detect OBI can prevent HBV transmission through blood transfusion.

## Introduction

Blood transfusion is an indispensable life-saving therapy for patient management; however, one of its adverse effects is the potential for transmitting infections. Hepatitis B virus (HBV) is the most frequent transfusion-transmitted viral infection.^1^ In sub-Saharan Africa, the median risk of HBV infection following blood transfusion is 4.3/1000 units^2^ and the risk from blood screened by enzyme immunoassay for recipients younger than ten years old is 1:326.^3^ Due to its public health burden, tackling HBV is a global health strategy for achieving the 2030 Agenda for Sustainable Development.^4^ When an individual has an acute-phase infection, the first viral antigen to appear in the blood is hepatitis B surface antigen (HBsAg), which persists even in the chronic phase but is undetectable when the virus is cleared from the blood. Thus, HBsAg is the HBV infection detection marker. The HBV genome is enclosed within a ‘core particle’ made of hepatitis B core protein or antigen (HBc). While the host is clearing the HBsAg by developing HBsAg antibodies (anti-HBs), the host also produces HBc immunoglobulin (antibody) M.

During this period, known as the ‘window period’, the host is infectious, and the serological marker for infection is anti-HBcIgM,^5^ as the period is associated with undetectable levels of both HBsAg and anti-HBsAg. Blood donors at this infection stage (tail end carriers) can transmit HBV.^6^ Progression of HBV infection to the chronic state is associated with the presence of both Immunoglobin G (IgG) and Immunoglobin M (IgM) anti-HBc against HBc. The natural course of infection in persons with chronic HBV infection could terminate as ‘inactive carriers’ with occult HBV infection (OBI), defined as the existence of low-level HBV DNA in the serum (< 200 IU/mL), cells of the lymphatic (immune) system, or hepatic tissue of patients with serological markers of the previous infection (anti-HBc or anti-HBs positive) and the absence of serum HBsAg.^7,8^ Hence, positive anti-HBc is considered a key OBI marker.^9^ Most blood bank screening panels consist of HBsAg and anti-HBc total (IgM + IgG).^10^ The anti-HBc total (IgM + IgG) is used to detect both previous and current HBV infection.

The HBsAg, anti-HBc, and HBV nucleic acid tests (NAT) are often used in blood banks in high-income countries to detect infection, during the window period, OBI, and genetic and antigenic viral variants, thus ensuring optimal blood safety levels. A substantial percentage of blood recipients are likely to be the immunosuppressed, women, and children.^11^ Transfusion of blood from OBI donors to pregnant women can increase vertical transmission of viral hepatitis. Vertically infected babies are prone to long-term complications of HBV infection, such as liver disease. Sadly, the University College Hospital (UCH) blood bank in Ibadan, Nigeria, cannot detect HBV infection in blood donors with undetectable HBsAg. In the UCH blood bank, as in many developing countries, most blood units are from replacement donors, against the recommended voluntary blood donors for safe blood supply. Replacement blood donors are family members or friends who donate blood to replace blood transfused relatives, in our setting pregnant relatives. Thus, the inability to detect HBV infection in blood donors with undetectable HBsAg implies a possible risk of post-transfusion hepatitis from anti-HBc-positive individuals. Therefore, it is imperative to determine the anti-HBc positivity status of replacement donors who constitute the most significant proportion of our blood donors^5,10^ as with Nigerian hospital-based blood banks.^12^ Anti-hepatitis B core antibody tests and NAT are excluded in our blood donor algorithm; therefore, this study aimed to assess the risk factors for positive anti-HBc in donors and the anti-HBc status of replacement donors to improve blood safety.

## Methods

### Ethical considerations

The University of Ibadan/University College Hospital (UI/UCH) ethics review team approved this research (19/0204). All recruited participants gave written informed consent. The confidentiality of participants was maintained by coding the samples, and only authorised personnel had access to participants’ identities.

### Study location and participants

The research was conducted at the blood bank of UCH in Ibadan, Nigeria, an 850-bed hospital with 163 examination couches, excluding private suites. This blood bank collects, stores, and processes blood. The target population were replacement blood donors whom the patient or patient’s relatives recruited to replace blood used. Only donors who presented blood donation forms indicating the patient on whose behalf blood was being donated were eligible. All donors were recruited from April 2019 to May 2019.

In our blood bank, donors were allowed to donate blood if: donors were between 18 and 65 years, weighed 50 kg and above, passed the copper sulphate test and the verbal questioning stage, and were negative for transfusion-transmitted infections, including syphilis, HIV, HBV and hepatitis C virus. Donors were excluded from this study if they: voluntarily came to donate blood, had a tattoo (exclusive of tribal marks obtained from infancy), failed the copper sulphate screening test or failed routine eligibility questioning by blood bank staff (which includes questions about previous blood donations, breastfeeding and menstruation status for female donors, presence of any health condition such as hypertension or diabetes, and HIV and hepatitis C virus status). In this study, a questionnaire was administered to include consecutive potential replacement blood donors who had passed the copper sulphate test and blood bank staff eligibility oral questioning stage. The questionnaire was translated into the native language (Yoruba) for non-English-speaking participants and collected the patient’s demographic characteristics and knowledge of and risk factors of HBV. Four questions tested HBV knowledge with ‘Yes’ or ‘No’ questions. Each ‘Yes’ response scored 1, while each ‘No’ response scored 0. Knowledge was graded from 0 to 4 based on increasing correct responses, that is, 1 = only one yes, 2 = two yeses, 3 = three yeses and 4 = four yeses.

### Sample collection

Six millilitres of venous blood were collected from each donor into sterile plain bottles labelled with the donor’s identity number. The blood was allowed to stand for 45 min, and then the serum was separated by centrifugation daily. The serum was then stored in two aliquots at –80 °C. The samples were thawed to room temperature for each laboratory analysis. The serological tests were performed a week after collecting samples, and the polymerase chain reaction (PCR) test was carried out in July 2019, two months after the collection of samples.

### Serology

The blood bank’s screening algorithm identifies a blood donor as HBV-positive using HBsAg rapid diagnostic test (RDT) kit and a conventional semi-automated enzyme-linked immunosorbent assay (ELISA). The Bio-Check Rapid kit test (Bio-Check, San Francisco, California, United States) employs lateral chromatographic flow, while the Monolisa™ HBs Ag ULTRA ELISA kit (Bio-Rad, Marnes-la-Coquette, France) employs a qualitative one-step solid-phase enzyme-linked immunoassay technique of the ‘sandwich’ type. Each test kit came with positive and negative controls used in each assay. First, the rapid test was administered to all donors pre-donation. Donors who were HBsAg-negative by the rapid test were further tested with the semi-automated ELISA test. Then, using one of the two aliquots of sera stored, all samples were tested further for anti-HBc using the HBcAb ELISA Kit (Melsin Medical Co., Ltd, Changchun, China). The anti-HBc assay measures the total anti-HBc, including the IgG and IgM HBc antibodies.

### Hepatitis B virus DNA detection

Sera from blood donors that were anti-HBc positive were tested for HBV DNA with primers targeting the HBV Pre-S gene in a semi-nested PCR protocol as described below. Briefly, the total HBV DNA was extracted from the samples using a DNA extraction method previously described by Wang et al.^12^ The HBV Pre-S gene was amplified in a semi-nested PCR using three primers. The 979 (5ʹCAAAAGACCCACAATTCTTTGACATACTTTCCAA3ʹ) and SF (5ʹGTGTCTTGGCCAAAATTCGCAGT3ʹ) primers were used in the first PCR run, while the primers 979 and MC-F (5ʹTCGGATCCGGTATGTTGCCCGTTTGTC3ʹ) were used in the second PCR round. The cycling conditions used are as follows: 95 °C for 5 min; 40 cycles of 95 °C for 30 s, 60 °C for 45 s, and 72 °C for 45 s; and a final extension of 72 °C for 7 min. The amplicon of ~550 base pairs was analysed by gel electrophoresis using 2% (weight/volume) agarose gel and stained with SYBR Stain (Jena Biosciences, Jena, Germany). Amplicons were visualised in an ultraviolet illuminator. The negative control was a previously known HBV-negative sample, while the positive control was a previously confirmed HBV DNA-positive sample. Occult HBV infection was defined as HBV DNA-positive, and serology HBsAg-negative and anti-HBc positive.

### Data analysis

Statistical Package for Social Sciences version 20 (IBM Corp., Chicago, Illinois, United States) was used. Frequencies and percentages in tables or charts were used to present the demographic characteristics, risk factors for HBV infection among donors, and hepatitis B screening results for participants. Means and standard deviations were used to summarise continuous variables such as the age of participants. Odds ratios (ORs) and 95% confidence intervals (CIs) were calculated to test for the association between risk factors and anti-HBc positivity. The Chi-square was also used to test the association between anti-HBc, socio-demographic features and risk factors of the participants. The statistical significance was set at *p* < 0.05.

## Results

### Socio-demographic and risk factors for acquisition of hepatitis B virus infection from blood donors

The 274 participants included in the study ranged from 18 to 62 years (mean age of 32.0 ± 8.86 years). Approximately half of the participants, 157 (57.3%), were married, and 25 (9.1%) had multiple sexual partners, while 14 (5.1%) had been previously treated for a sexually transmitted disease (Table 1). Twenty-eight (10.2%) participants had scarification or tribal marks, 87 (31.8%) shared sharp objects (pedicure, manicure and use of razor blade), and 9 (3.3%) had previously used sex performance enhancement recreational drugs. Furthermore, 18 (6.6%) participants had been previously transfused with blood, 7 (2.2%) had jaundice in the last year, and 57 (20.8%) had been asked to do a test for hepatitis B before coming to donate blood. Participants’ HBV knowledge scores were: zero, 120 (46.9%); one, 29 (11.3%); two, 21 (8.1%); three, 57 (22.3%); and four, 29 (11.3%).

**TABLE 1:** Demographic characteristics of 274 blood donors in Ibadan, Nigeria, between April 2019 and May 2019.

Variables	Frequency	Percentage
**Age (in years)**		
18–25	81	23.4
26–35	142	41.0
36–45	96	27.9
46–55	22	6.4
56–65	3	0.9
**Sex**		
Male	237	86.5
Female	37	13.5
**Marital status**		
Married	157	57.3
Single	116	42.3
Widowed	1	0.4
**Educational level**		
Primary	11	4.0
Secondary	91	33.2
Tertiary	172	62.8
**Occupation**		
Vocational	40	14.6
Unskilled	22	8.0
Skilled	127	46.4
Professional	85	31.0

### Risk factors for hepatitis B virus and anti-hepatitis B core protein or antigen positivity

**FIGURE 1:**
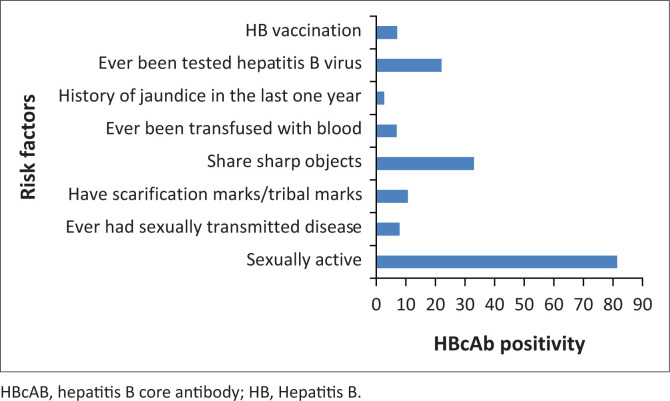
Risk factors for Hepatitis B virus infection among 133 donors with positive anti-hepatitis B core protein or antigen in Ibadan, Nigeria, between April 2019 and May 2019.

A total of 133 blood donors were anti-HBc positive. The proportion of participants aged 18–35 years with a positive anti-HBc (42.2%) was lower than the proportion of participants above 36 years with a positive anti-HBc (61.8%). Donors showed a declining prevalence of anti-HBc with an increasing HBV knowledge score (*p* = 0.046). The odds of being HBcAb positive were 0.35 times less likely among blood donors who had a knowledge score of 4 than blood donors who had a knowledge score of 0 (95% CI: 0.14; 0.84). All 9 participants who used recreational drugs had a positive anti-HBc compared to 117/252 (46.8%) of participants who did not use (*p* = 0.001). In contrast, there was a lower proportion of participants who shared sharp objects with positive anti-HBc, 34 (39.1%), compared to 96 (52.9%) for those who did not share (Table 2). The sharp objects shared included blades and sharps used for a pedicure, manicure, beauty treatment, and shaving hair. The age, gender, educational level, number of sexual partners, history of sexually transmitted disease transfusion and jaundice did not show any significant statistical relationship to anti-HBc positivity. Thirteen donors had prior HBV vaccination. Five (33.3%) of the HBV vaccinated donors were positive for anti-HBc, while 10 (66.7%) were negative. However, the difference was not statistically significant. The odds of being HBcAb positive were 2.22 times more likely among blood donors over 35 years old than those aged 18–35 years (95% CI: 1.32; 3.72). The odds of being HBcAb positive were 0.35 times less likely among blood donors who had a knowledge score of 4 than blood donors who had a knowledge score of 0 (95% CI: 0.14; 0.84). The relative risk (RR) of blood donors being HBcAb positive was 2.14 times higher among blood donors who had ever used recreational drugs before or during sex than in blood donors who never used recreational drugs before or during sex (95% CI: 1.88; 2.43). There is a 2.23 times higher likelihood of anti-HBc positivity for blood donors who had ever been transfused with blood. Blood donors who shared sharp objects were 0.57 times less likely to be HBcAb positive compared to donors who do not share sharp objects (95% CI of OR: 0.34; 0.96). The comparison of the different risk factors with HBcAb is shown in Figure 1.

**TABLE 2:** Comparison of hepatitis B virus risk factors among anti-hepatitis B core-positive and -negative blood donors in Ibadan, Nigeria, between April and May 2019.

Variables	HBcAB				*p*	OR	95% CI of OR
	Negative		Positive				
	*n*	%	*n*	%			
**Age**							1.322; 3.724
18 to 35 years	107	57.8	78	42.2	-	-	-
Over 35 years	34	38.2	55	61.8	0.02	2.219	-
**Sex**							0.443; 1.776
Male	121	51.1	116	48.9	-	-	-
Female	20	54.1	17	45.9	0.734	0.887	
**Ethnicity (*n* = 273)**							0.049; 1.101
Yoruba	127	51.2	121	48.8		-	0.115; 4.260
Igbo	9	81.8	2	18.2		0.233	1.035; 68.14
Hausa	3	60.0	2	40.0	0.012	0.700	-
Others	1	11.1	8	88.9		8.397	-
**Occupation (273)**							
Vocational	19	47.5	21	52.5	-	-	-
Unskilled	10	45.5	12	54.5	-	1.086	0.382; 3.083
Skilled	66	52.0	61	48.0	0.815	0.836	0.410; 1.703
Professional	46	54.8	38	45.2	-	0.747	0.351; 1.590
**Educational Level**							
Primary	8	72.7	3	27.3	-	-	-
Secondary	41	45.1	50	54.9	0.152	3.252	0.810; 13.05
Tertiary	92	53.5	80	46.5	-	2.319	0.595; 9.037
**Hepatitis B is caused by a virus (*n* = 252)**							
No	59	46.1	69	53.9	-	-	-
Yes	71	57.3	53	42.7	0.076	0.632	0.388; 1.050
**Hepatitis B virus can be sexually transmitted (*n* = 241)**							0.294; 0.848
No	67	45.3	81	54.7	0.010	0.499	-
Yes	58	62.4	35	37.6	-	-	-
**Hepatitis B virus can be transmitted through sharp object (*n* = 232)**							0.301; 0.880
No	64	46.0	75	54.0	0.015	0.515	-
Yes	58	62.4	35	37.6	-	-	-
**Hepatitis B cannot be cured (*n* = 224)**							0.648; 2.326
No	97	55.1	79	44.9	-	-	-
Yes	24	50.0	24	50.0	0.529	1.228	-
**Knowledge score (*n* = 256)**							
0	57	47.5	63	52.5	-	-	0.431; 2.183
1	14	48.3	15	51.7	-	0.940	0.682; 4.800
2	7	33.3	14	66.7	0.046	1.810	0.348; 1.243
3	33	57.9	24	42.1	-	0.658	0.142; 0.839
4	21	72.4	8	27.6	-	0.345	-
**Sexually active**							0.619; 1.978
No	32	54.2	27	45.8	-	-	-
Yes	109	50.7	106	49.3	0.630	1.106	-
**Number of sex partners (*n* = 216)**							0.592; 3.170
1	99	51.8	92	48.2	-	-	-
2	11	44.0	14	56.0	0.461	1.370	-
**Ever had sexually transmitted disease (216)**							0.474; 4.225
No	104	52.8	98	47.2	-	-	-
Yes	6	42.9	8	57.1	0.532	1.415	-
**Have Tattoo/scarification marks/tribal marks**							0.668; 3.237
No	129	52.4	117	47.6	-	-	-
Yes	12	42.9	16	57.1	0.336	1.470	-
Ever used recreational drugs before or during sex*							1.880; 2.430
No	141	53.2	124	46.8	-	-	-
Yes	0	0.0	9	100	0.001	2.137	-
**Share sharp objects**							
No	88	47.1	99	52.9	-	-	0.340; 0.957
Yes	53	60.9	34	39.1	0.033	0.570	-
**Ever been transfused with blood**							0.813; 6.128
No	135	52.7	121	47.3	-	-	-
Yes	6	33.3	12	66.7	0.111	2.231	-
**History of jaundice in the last one year**							0.313; 6.496
No	138	51.7	129	48.3	-	-	-
Yes	3	42.9	4	57.1	0.716	1.426	-
**Ever been tested hepatitis B virus**							0.480; 1.548
No	110	50.7	107	49.3	-	-	-
Yes	31	54.4	26	45.6	0.619	0.862	-
**Hepatitis B vaccination**							
No	133	51.0	128	49.0	-	-	-
Yes	8	61.5	5	38.5	0.456	1.649	0.207; 2.037

Note: 216 respondents were sexually active so only 216 donors responded to queries on sexual history. The other variables that were not equal to 274 were due to no responses from the respondent.

HBcAB, hepatitis B core antibody; CI, confidence interval; OR, odds ratio.

* , Relative risk (RR) was computed.

### Occult hepatitis B virus infection

Of the 274 blood donors, 42 (15.3%) cases were HBsAg-positive by both or either RDT or ELISA: 9 (3.28%) by both RDT and ELISA, 6 (2.19%) by RDT only, and 27 (9.85%) by ELISA only; 232 (84.7%) were HBsAg-negative (RDT and ELISA negative). Almost half (133/274; 48.5%) of the donors were anti-HBc positive, while 26/274 (9.5%) donors were HBsAg-positive and anti-HBc positive. Among the 259 participants who tested negative for hepatitis B by RDT, 121 (46.7%) had a positive anti-HBc result, while 110 (46.2%) of the 238 participants who tested negative for HBsAg by ELISA test had a positive anti-HBc (Table 3).

**TABLE 3:** Patterns of anti-hepatitis B core results for rapid and enzyme-linked immunosorbent assay tests for hepatitis B surface antigen and relationship of the two hepatitis B surface antigen detection assays in Ibadan, Nigeria, between April 2019 and May 2019.

Variables	Negative		Positive	
	*n*	%	*n*	%
**Anti-HBc**				
HBsAg rapid test				
Negative	138	53.3	121	46.7
Positive	3	20.0	12	80.0
HBsAg ELISA				
Negative	128	53.8	110	46.2
Positive	13	36.1	23	63.9
HBsAg-Negative by ELISA and RDT (*n* = 232)	125	53.9	107	46.1
**HBsAg RDT**				
HBsAg ELISA				
Negative	232	97.5	6	2.5
Positive	27	75.0	9	25.0

ELISA, enzyme-linked immunosorbent assay; RDT, rapid diagnostic test; HBc, hepatitis B core; HBsAg, Hepatitis B surface antigen.

Of the 232 blood donors that were HBsAg-negative by any method, 107 (46.1%) were positive for anti-HBc. However, only one case was anti-HBc positive, HBsAg-negative and HBV DNA-positive (0.93%).

## Discussion

This study on HBc antibodies among replacement blood donors, who form a significant proportion of blood donors in Nigeria, showed that 48.8% were positive for total anti-HBc (IgG and IgM), and over 60.0% of the donors had a tertiary education level, with 47.0% of the donors having an HBV infection knowledge score of 0. In addition, the risk factors for HBV acquisition among anti-HBc-positive donors were being older than 35 years, having poor knowledge of the HBV transmission route, and the use of sex enhancement recreational drugs.

An inconsistent positivity rate was observed when RDT and ELISA were used to screen for HBsAg among blood donors. This incongruity in the results suggests that it is better to use both methods in screening blood for transfusion to reduce the escape of HBV-positive blood into the transfusion pool, especially when HBV DNA screening is not in use.

Testing for anti-HBc has been used in blood transfusion in low HBV-endemic regions to minimise the incidence of post-transfusion hepatitis following transfusion of HBsAg-negative blood. The assay identifies chronically infected low-level HBV donors.^13^ The prevalence of anti-HBc (35.7%) among our HBsAg-negative blood donors is high. Other studies have reported lower anti-HBc prevalence in low and intermediate HBV-endemic regions and in some high HBV-endemic regions like India^14^ and North Africa.^15^ The higher prevalence of anti-HBc (35.7%) among the blood donors may be attributed to a higher number of blood donors who had current or past exposure to HBV since anti-HBc persists for life even though the HBV infection is resolved. The possibility of OBI should be considered in HBsAg-negative but anti-HBc-positive blood donors.

Occult HBV is more frequently diagnosed in anti-HBc-positive individuals than in anti-HBc-negative individuals.^16,17^ Occult HBV has been reported to range from 3.0% to 17.0% in Nigeria.^18,19^ The prevalence of anti-HBc antibody positivity among blood donors varies between and within countries, depending on the assay method. The anti-HBc test lacks specificity as test reagents reactivity varies between manufacturers, which may affect between-study comparisons.^20^ Depending on the assay type and screening algorithms, false-reactivity rates ranged between 16.0% and 75.0%.^21^ The confirmatory algorithm is complex. However, a positive anti-HBc in a high HBV-endemic region establishes a history of HBV infection and portends a high risk of OBI with the possibility of deferring the blood donor. Japhet et al. from Nigeria reported a lower anti-HBc prevalence of 13.0% and a higher prevalence of 19.6% HBsAg.^22^ The lower anti-HBc prevalence was probably caused by the IgM anti-HBc test used in that study, which detects acute infections but misses chronic HBV carriers, whereas the current study detected total anti-HBc (IgM and IgG). The shortcoming of the total anti-HBc study is that it includes all blood donors who had been exposed to HBV including those with resolved infection. This shortcoming may exaggerate the prevalence of HBV infection in high-prevalence regions, thereby shrinking the size of the eligible blood donor pool. Dhawan et al. observed a significantly higher anti-HBc (8.4% vs 6.9%) among replacement donors than among voluntary donors in India. However, a wide range of prevalence of anti-HBc positivity has been reported among Indian donors, and this has been attributed to the use of assays with varying sensitivities in different studies for screening the donors and the nature of the study population.^23^

This study suggests that donors who had ever used recreational drugs are likely to be positive for anti-HBc. Therefore, stringent blood donor screening criteria using a standard questionnaire that provides confidentiality for donors in addition to educational materials to eliminate donors who had ever used recreational drugs may significantly reduce HBV-positive donors irrespective of whether they are voluntary or replacement blood donors. Comparing this study with other studies that investigated total anti-HBc in Nigeria, the prevalence of 48.5% is higher than 32.5% by Ogunfemi et al. in Ilorin,^24^ but lower than 60.7% and 90.0% from the studies by Akinbami et al. in Lagos^18^ and Ojo et al. in Ife.^25^ The 90.0% prevalence was reported pre-HBV vaccination. Levels of anti-HBc have been shown to decrease in the donor population; this could be attributed to acquired immunity from vaccination and increased awareness.^26^ Similarly, in Germany, the trend for anti-HBc-positive status was 1.17% in 2007 but decreased to 0.72% in 2015.^27^ Summarily, the variations in prevalence have been associated with the different assays used, study design and HBV endemicity. Some studies investigated anti-HBc in HBsAg-negative donors, while some studies investigated in both HBsAg-negative and HBsAg-positive donors.^23,25,26,27^

Total anti-HBc+/HBsAg- donors of 107 (46.1%) in our study is higher than IgM anti-HBc+/HBsAg- donors of 49 (18.1%) and 20 (4.4%) for Maiduguri^28^ and Ile-Ife,^29^ Nigeria. The higher anti-HBc+/HBsAg- donors in our study could suggest a higher potential for HBV blood transmission from donors. However, this may not be the case because high anti-HBc prevalence does not always imply high OBI frequency.^27^ Although other studies reported a lower prevalence of anti-HBc in some Nigerian blood centres, the anti-HBc prevalence in those blood centres is still significant to cause an increase in donor deferral rate and reduce blood supply if anti-HBc testing is added to the blood donor screening algorithm. The anti-HBc test included in screening blood donors in HBV non-endemic countries is not routinely used in HBV-endemic countries because many blood products would be discarded due to positive result even though most of the blood would be safe for transfusion.^20^

Total anti-HBc-positivity was significantly higher among donors older than 35 years compared to younger donors, which is contrary to the statistically non-significant difference observed in Ilorin, Nigeria.^23,24^ Studies from Bangladesh, Italy, and Korea also reported high anti-HBc-positive status in older donors, which was attributed to the acquisition of HBV infections in earlier years when HBV was highly endemic.^30,31,32^ Although the prevalence of HBV infection is still high in Nigeria, declining rates have been reported.^33^ This study suggests that donors younger than 35 years may be less likely to have HBV infection or be an occult HBV carrier. Occupation and educational level did not significantly affect the anti-HBc status of the donors. However, a good knowledge of the virus transmission was associated with a lower anti-HBc-positive rate, which suggests that ignorance about the virus increases vulnerability to infection. Therefore, health education to increase awareness of HBV transmission and prevention could reduce infection among the future donor population. The finding of lower anti-HBc antibodies among those who share sharp objects in salons may not have any obvious explanation, but Olayinka et al. reported in a study in Nigeria that public barbing salon clipper cuts, manicure and pedicure cuts, and scarification were not significantly associated with the presence of HBsAg.^34^ All blood donors who used recreational drugs were anti-HBc positive. Blood donors who use recreational drugs and are positive should be identified and permanently deferred from donating blood. Surprisingly, five blood donors who had HBV vaccination were anti-HBc positive. Vaccine recipients who develop anti-HBc might not have responded to the vaccine. It may also be indicative of HBV infection with escape mutants.^20,33^ The anti-HBsAg (anti-HBs) titre of the vaccinated donors was not determined, so we could not confirm if the vaccination provoked an immune response in them. Sharing of sharp objects, being sexually active, the number of sex partners and unprotected sex evidenced by those with an sexually transmitted disease are risk factors for HBV infection^35,36^ but were not evident in our study. This might be due to the targeted study population and the study’s small sample size. The finding in this study does not rule out the danger associated with collecting blood from donors with risk factors. Recruiting donors with risk factors is strongly discouraged, especially where exhaustive screening tests are not available.

The screening of the donors with anti-HBc-positive and HBsAg-negative sera for HBV DNA showed that occult infection was present in one of the 107 donors (0.93%). The presence of HBV DNA in 0.93% of our serologically screened donors suggests that there is a potential for transmission of hepatitis B to blood recipients. This is higher than 0.56% of blood donors in Cameroon^35^ and 0.5% in Ghana,^36,37^ but less than 11.54% – 14.5% reported in blood donors in Egypt.^37,38^ This study establishes that HBV infection may occur among recipients of blood from donors that are isolated anti-HBc positive. It might be difficult to sustain the blood supply if all the 48.5% of the blood donors who have anti-HBc in their blood are deferred or rejected. Implementing anti-HBc testing could make blood safer. Since exclusion of all anti-HBc-positive donors may reduce the availability of safe blood in the blood banks of countries with high HBV prevalence, and screening all blood donors with NAT to determine the HBV DNA status may be too expensive in resource-limited regions, an algorithm that attempts to detect occult HBV is being suggested. The complementary use of both RDT and ELISA for HBsAg screening of blood donors should continue for maximum identification of HBsAg-positive donors. The recommended algorithm includes testing all HBsAg-negative blood (by both RDT and ELISA) for antibodies to HBc and subjecting anti-HBc-positive blood to NAT to identify potential infectious blood units. This will improve the safety of the blood supply and reduce costs while capturing samples that are likely to have undetected threats.

### Limitations

The confirmatory algorithms for true positivity of anti-HBc include secondary testing with an alternative ELISA, testing for anti-HBs, anti-HBe or HBeAg. The most frequently used is anti-HBs. However, this study did not investigate the anti-HBs titre of the participants. This might affect the significance of the observed anti-HBc and HBV DNA positivity in the donor population.

### Conclusion

Almost half of the donors in this study were anti-HBc positive, suggesting that a large proportion of the donors had been exposed to HBV. Moreover, donors who were anti-HBc positive and HBsAg-negative could have HBV DNA. We propose a testing algorithm that can be utilised in HBV-endemic, resource-limited settings. All donors should be tested for HBsAg (by both ELISA and RDT); those who are HBsAg-negative should be further tested for anti-HBc, while only those who are both HBsAg-negative and anti-HBc positive should undergo mini-pool NAT. This algorithm optimises blood safety and prevents HBV transmission from infected blood units.
